# Roles of Twist1 in lipid and glucose metabolism

**DOI:** 10.1186/s12964-023-01262-6

**Published:** 2023-10-02

**Authors:** Liuyifei Huang, Yan Xing, Xiaoxuan Ning, Zhixiang Yu, Xiao Bai, Limin Liu, Shiren Sun

**Affiliations:** 1 Department of Nephrology, Xijing Hospital, The Fourth Military Medical University, Changle Road, No. 127 Changle West Road, Xi’an, Shaanxi China; 2 Department of Geriatrics, Xijing Hospital, The Fourth Military Medical University, Changle Road, No. 127 Changle West Road, Xi’an, Shaanxi China; 3 School of Medicine, Northwest University, 229 Taibai North Road, Xi’an, 710032 Shaanxi China

**Keywords:** Twist1, Lipid metabolism, Glucose metabolism, Adipose tissue, Fatty acid oxidation

## Abstract

The abnormal lipid and glucose metabolisms are linked to the metabolic disorders, tumorigenesis, and fibrotic diseases, which attracts the increasing attention to find out the key molecules involved in the lipid and glucose metabolism as the possible therapeutic targets on these diseases. A transcriptional factor Twist1 has been associated with not only the embryonic development, cancer, and fibrotic diseases, but also the regulation of lipid and glucose metabolism. In this review, we will discuss the roles and mechanisms of Twist1 in the obesity-associated white adipose tissue inflammation and insulin resistance, brown adipose tissue metabolism, fatty acid oxidation, and glucose metabolism in skeletal muscle to provide a rational perspective to consider Twist1 as a potential treatment target in clinic.

Video Abstract

**Supplementary Information:**

The online version contains supplementary material available at 10.1186/s12964-023-01262-6.

## Background

The metabolic dysregulation of lipids and glucose has been associated with metabolic disorders, tumorigenesis, and fibrotic diseases. It is urgent to discover the critical therapeutic targets for the clinical treatment which are involved in the lipid and glucose metabolism. Twist1, a class B member of basic helix-loop-helix (bHLH) transcriptional factor family [1, 2], contributes to a variety of fibrotic diseases [3]*,* including kidney fibrosis [4–7]*,* pulmonary fibrosis [8–10]*,* liver fibrosis [11]*, and* skin fibrosis [12]*.* The transcriptional functions of Twist1 are to dimerize with other bHLH members and bind to cis-regulatory elements for the signal transduction [13, 14]*.* The considerable importance of Twist1 as a zygotic gene has been established in the dorsal–ventral patterning, mesodermal differentiation, and subdivision of the mesoderm during early embryonic development [15, 16]*.* Apart from the physiological and biological roles in embryonic development and organogenesis, Twist1 has been associated with tumorigenesis, tumor progression, metastasis, stemness, and vasculogenic mimicry [17–24]. Importantly, more and more studies have linked Twist1 to lipid metabolism in adipose tissue.

Twist1 is highly expressed in the adipocytes of white adipose tissue and is elevated during adipogenesis of human pre-adipocytes [25]*.* Intriguingly, the expression level of Twist1 is decreased in obese patients and increased after weight loss [26]*.* Twist1 has also been found to associate with insulin resistance (IR) in adipocytes, which may provide us a new therapeutic direction towards diabetes and obesity [27]*.* In 2009 Twist1 was first found to be related to brown fat metabolism by forming a negative feedback loop of Peroxisome proliferator-activated receptor-γ coactivator (PGC)-1α / peroxisome proliferator activated receptor (PPAR)-δ to suppress PGC-1α mediated mitochondrial oxidative metabolism and uncoupling [28]*.* Furthermore, Twist1 has been associated with fatty acid oxidation (FAO). For example, Twist1 is indispensable for Th1 lymphocytes to survive when fatty acid oxidation is the only available metabolic pathway [29]*.* The role of Twist1 in renal fibrosis has also been recently elucidated by our group [30]. Hypoxia-induced activation of Twist1 resulted in mitochondrial dysfunction, intracellular lipid accumulation, and FAO defects in tubular epithelial cells (TECs), causing lipotoxicity and tubulointerstitial fibrosis (TIF). In glucose metabolism, Twist1 mainly participates in Warburg effect, a phenomenon that tumor tissues metabolize approximately tenfold more glucose into lactate than normal tissues under the aerobic conditions, thus promoting tumor metastasis and progression [31, 32]*.* However, the functions of Twist1 in amino acid metabolism are not thoroughly explored yet.

Despite the research on the roles of Twist1 in embryonic development, cancer, and fibrotic diseases to date, it is essential to further explore how Twist1 regulates lipid and glucose metabolism. In this review, we summarize the functions of Twist1 in lipid and glucose metabolism to provide the possible therapeutic strategy against these diseases.

### Twist1 in lipid metabolism

Twist1 has the highest abundance in the adipose tissue, which reached more than tenfold higher than other tissues in adult mice [28]*.* Adipose tissue is usually divided into white adipose tissue (WAT) and brown adipose tissue (BAT) [33]*.* White adipose tissue (WAT), as an energy storage center, is widely distributed in human body and is commonly categorized into the subcutaneous adipose tissue that mainly resides in the gluteal-femoral and abdominal region, and the visceral adipose tissue that is usually located in the omentum, mesentery, mediastinum, and epicardium [34]*.* Brown adipose tissue (BAT), predominantly located in the scapular of human newborns and rodents, is of momentous significance to neonates, whose temperature regulation center is not yet mature enough to defend themselves against the low temperature. However, BAT gradually decreases with age [35, 36]. When stimulated by low temperature, WAT underwent the browning process called adaptive thermogenesis, which renders WAT adopt the characteristics of BAT leading to the formation of beige adipose tissue [35, 36]*.* Since Twist1 hasn’t been probed in beige adipose tissue, in the following sections we will focus on the WAT and BAT.

#### Twist1 in White Adipose Tissue (WAT)

Obesity and insulin resistance are closely related to chronic inflammation which often parallels to the accumulation of overwhelming lipid in adipose tissue, presented by the alterations in inflammatory cells and inflammation markers, anomalous cytokine production, and the activation of inflammatory signaling pathways [37, 38]*.* A plenty of evidence suggests that inflammation is directly associated with insulin resistance [39]*.* For example, insulin-resistant obese individuals exhibit more severe adipose tissue inflammation than that in insulin-sensitive obese individuals [40]*.* Adipose tissue is a sophisticated and functionally active secretory organ both sending and receiving the signals that mediates metabolic homeostasis, insulin sensitivity, inflammation, and immunity [41]*.* A great many molecular signaling pathways have been involved in the crosstalk between inflammation and metabolism. For instance, the NF-κB pathway and the c-Jun N-terminal kinase (JNK) pathway are of tight relations to the proinflammatory effects of obesity and insulin resistance. Obesity is associated with WAT inflammation, which is characterized by an increased infiltration of macrophages and elevated secretion of several cytokines and chemokines from both macrophages and adipocytes, such as tumor necrosis factor-α (TNF-α), interleukin-6 (IL-6), and monocyte chemotactic protein-1 (MCP-1) [40]*.* Loss-function of NF-κB pathway, JNK pathway, and pro-inflammatory signaling molecules inhibits inflammatory signaling and disrupts the obesity related insulin resistance in obese mice [38, 42, 43]*.* Therefore, a better understanding of how obesity induced inflammation plays a pathogenic role in the development and progression of insulin resistance could remarkably contribute to the solution or possible treatments of these metabolic disorders.

Previous studies have reported that Twist1 is most abundantly expressed in the WAT and BAT in adult mice, and that its expression is negatively correlated with the homeostasis model assessment of insulin resistance (HOMA-IR) and adipocyte size in human [28]*.* Interestingly, the controversial discoveries were found in animal studies, suggesting that Twist1 transgenic mice were more prone to high-fat diet-induced obesity phenotype, whereas Twist1 heterozygous knockout mice displayed obesity-resistance phenotype [28]*.* In humans, Twist1 gene expression level is decreased in obesity and increased following surgical or caloric restriction weight loss in both subcutaneous adipose tissue (SAT) and visceral adipose tissue (VAT) despite of its differential expression in the SAT and VAT [44]*.* Considering the differences in inflammation between SAT and VAT, it is conceivable that Twist1 may have anti-inflammatory effects that are more prominent in SAT compared to VAT [44]*.* Twist1 expression level was reduced by more than 50% in isolated adipocytes from obese subjects. Consistently, Twist1 was upregulated during 3T3-L1 preadipocyte differentiation without crippling lipid formation and was negatively correlated with the obesity development [45]*.* Thus, Twist1 could be one of the key factors controlling inflammatory gene expression in human adipocytes.

One potential underlying mechanism is the positive regulation of GPS2 and SMRT expression by Twist1. The silencing of Twist1 in adipocytes caused downregulation of GPS2 and SMRT expression and upregulation of inflammatory genes expression, such as IL-6 and IL-8 [46]*.* Pettersson and colleague suggested that Twist1 might play a role in the inflammation of human WAT by regulating the expression and secretion of inflammatory adipokines via direct transcriptional effects in white adipocytes [25]. Twist1 was detected majorly in WAT with considerably higher expression in adipocytes than in other cell types of human WAT, by contrast Twist2 expression presented similar levels in all examined tissues. Moreover, the Twist1 expression in human WAT was relatively low under the circumstances of obesity and insulin-resistance, which may be mediated by an increased sensitivity to the proinflammatory effect of TNF-α [26]*.* In human differentiated adipocytes, the expression and secretion of the inflammatory factors TNF-α, IL-6, and MCP-1 were downregulated by Twist1 knockdown since Twist1 directly bound to the E-boxes in the promoters of these genes and was required for their basal transcription [25]*.* However, in another study by the same group showed that TNF-α and MCP-1 secretion from WAT was increased after Twist1 silencing in human adipocytes, which was accompanied by the upregulation in mRNA levels of RelA and TNF-R1, two important components that can enhance the sensitivity to TNF-α [26]*.* The further clinical data showed that the mRNA levels of TNF-α, IL-6, and MCP-1 were significantly increased in the obese individuals, which suggested that the condition of low Twist1 expression with elevated TNF-α levels may have the pathological rather than the beneficial effects. In the environment where TNF-α is absent or at low levels, Twist 1 is required for the basal transcription of cytokines and chemokines [26]*.* Besides, Twist1 heterozygous knockout mice have increased circulating levels of the inflammatory cytokines TNF-α, IL-1β, and IL-6. The opposite results of gene silencing experiments between knockout mice and adipocytes may attribute to the species-specific differences [47]*.* These contradictory results provide us with a novel idea that the role of Twist1 in adipose tissue may differ depending on the species and the status quo. Hence, further research is still needed to determine the precise mechanism by which Twist1 regulates the inflammatory factors in WAT.

Furthermore, the individuals with weight loss and with relieved adipose tissue inflammation demonstrated significantly increased expression of Twist1, SMRT, and GPS2, as well as the decreased expression of inflammatory cytokines, such as IL-6, IL-8, and MCP-1. PPARγ acted as an upstream regulator of Twist1/SMRT/GPS2 cascade and repressed the inflammatory genes in human adipocytes [46]*.* Pioglitazone, an antidiabetic and anti-inflammatory PPARγ agonist, restored the expression of Twist1, GPS2, and SMRT in adipose tissue in diabetic obese patients [46]*.* In addition, Twist1 was found elevated in high glucose/insulin stimulated IR 3T3-L1 adipocytes and IR C57/BL6J mouse model induced by high fat diet (HFD), and Twist 1 silencing attenuated IR in vitro and in vivo by regulating the downstream IRS/PI3K/AKT/GluT4 pathway to relieve the mitochondrial dysfunction in IR cells [27]*.* It is still unknown if Twist1 directly interacts with the known signal pathways associated with inflammation, such as NF-κB pathway and JNK pathway. The analysis of transcriptome and proteome could be utilized to screen the altered inflammatory factors through other transcription factors. Taken together, Twist1 is a novel transcription factor with profound implications in the development of obesity-associated WAT inflammation and insulin resistance, which provides a prospective mechanism linking Twist1 expression with the obesity-associated diseases.

#### Twist1 in Brown Adipose Tissue (BAT)

Brown adipocytes (BAs) are known as the ability of thermogenesis, which is defined by their large quantity of mitochondria and dense vascularization [36, 37]. The expression level of Twist1 was increased in brown fat mature adipocytes compared to preadipocytes, although no significant effect of Twist1 on adipogenic differentiation was observed. The transcriptional activities of two BAT-specific PGC isoforms (PGC-1α and NT-PGC-1α) were differentially influenced by Twist1 [48]*.* The N-terminal region of Twist1 directly interacted with the Twist1-binding domain on C-terminal region (aa 353–797) of PGC-1α to inhibit the transcriptional activity of PGC-1α [28], whereas the Twist1-binding domain was absent in NT-PGC-1α resulting in no transcriptional effects on NT-PGC-1α by Twist1 [48]*.* Furthermore, PPARδ bound to the Twist1 promoter and activated Twist1 expression in both brown adipocytes and animal models. In the presence of PPARδ both Twist1 and PGC-1α were associated with the PPAR-binding site of the mitochondrial uncoupling protein 1 (UCP1) promoter, however, when PPARδ was absent PGC-1α was unable to interact with the UCP1 promoter, which in turn led to the loss-function of Twist1 on UCP1 promoter [28]*.*

Twist1 acted as a pivotal negative regulator of mitochondrial oxidative metabolism and uncoupling mediated by PGC-1α in BAT. Albeit the other isoform of PGC-1α, NT-PGC-1α, regulated the transcriptional activity of mitochondrial and thermogenic genes through facilitating the activity of nuclear receptors such as PPARs, the expression levels of CPT1β, UCP1, and ERRα were unchanged in the absence of Twist1 [48]*.* Overexpression of Twist1 in brown adipocytes not only caused an immense reduction of PGC-1α target genes CPT1β, UCP1, and ERRα, which are involved in oxygen consumption, but also decreased mitochondrial fatty acid oxidation and uncoupling [28, 48]*.* Consistently, the ablation of Twist1 increased the mitochondrial DNA content and catalyzed mitochondrial biogenesis. In Twist1 transgenic mice, similarly, the expression levels of UCP1 and the genes related to fatty acid oxidation (FAO) were remarkably decreased in the BAT. Therefore, Twist1 disparately regulates PGC-1α and NT-PGC-1α activities. Yet no differences of FAO-related gene expression were found in the WAT due to low abundance of PGC-1α in WAT.

In brown fat metabolism, transcription factors are of great significance, including PPARγ, PPARα, ERRα, NRF1, and PGC-1α [48]*.* Interestingly, Twist1 and PPARγ protein levels were even more upregulated in mature 3T3-L1 adipocytes than preadipocytes [49]*.* Downregulating Twist1 enhanced the expression of PPARγ and influenced the secretion of multifarious adipokines, interleukins, growth factors, chemokines, and their receptors [45]*.* Nevertheless, the contradiction appears concerning the crosstalk between Twist1 and PPARγ expression. For example, the lentivirus-mediated Twist1 overexpression upregulated the PPARγ expression in the 4-day post-differentiated preadipocytes [45]*,* and downregulated the PPARγ levels in mature adipocytes, which is likely due to the differences between two experimental cell lines [49]*.* Conversely, the expression level of Twist1 was upregulated by the PPARγ agonist or downregulated by the PPARγ antagonist. In humans, it has been discovered that PPARγ agonist pioglitazone treatment increased Twist1 expression in adipose tissue through the Twist1/SMRT/GPS2 cascade in diabetic obese patients [46]*.*

Besides, microRNAs also interact with Twist1 to regulate brown fat metabolism. For instance, miR-337-3p was identified to express sevenfold higher during the maturation process of murine brown adipocytes compared with that during pre-differentiation. Overexpression of miR-337-3p bolstered brown fat metabolism via stimulating a reduction in Twist1 and increasing the expression levels of browning markers, mitochondrial markers, and the downstream UCP1, thereby enhancing the browning of adipocytes [50]*.* In sum, Twist1 is an innovative regulator of BAT metabolism by regulating PGC-1α and PPARδ. The function of Twist1 in brown adipose metabolism as well as the underlying mechanisms are unclear. Metabolic Chamber analysis is potential to investigate if Twist1 changes the energy expenditure in vivo. It is also needed to identify more microRNAs that regulate Twist1 mediated brown adipose metabolism. The application of microRNAs aiming at Twist1 will provide a potential strategy to modulate Twist1 in humans. More evidence is necessary to understand the mechanisms by which Twist1 plays a role in regulating PPARγ expression as well as WAT browning.

#### Twist1 in Fatty Acid Oxidation (FAO)

**Fig. 1:**
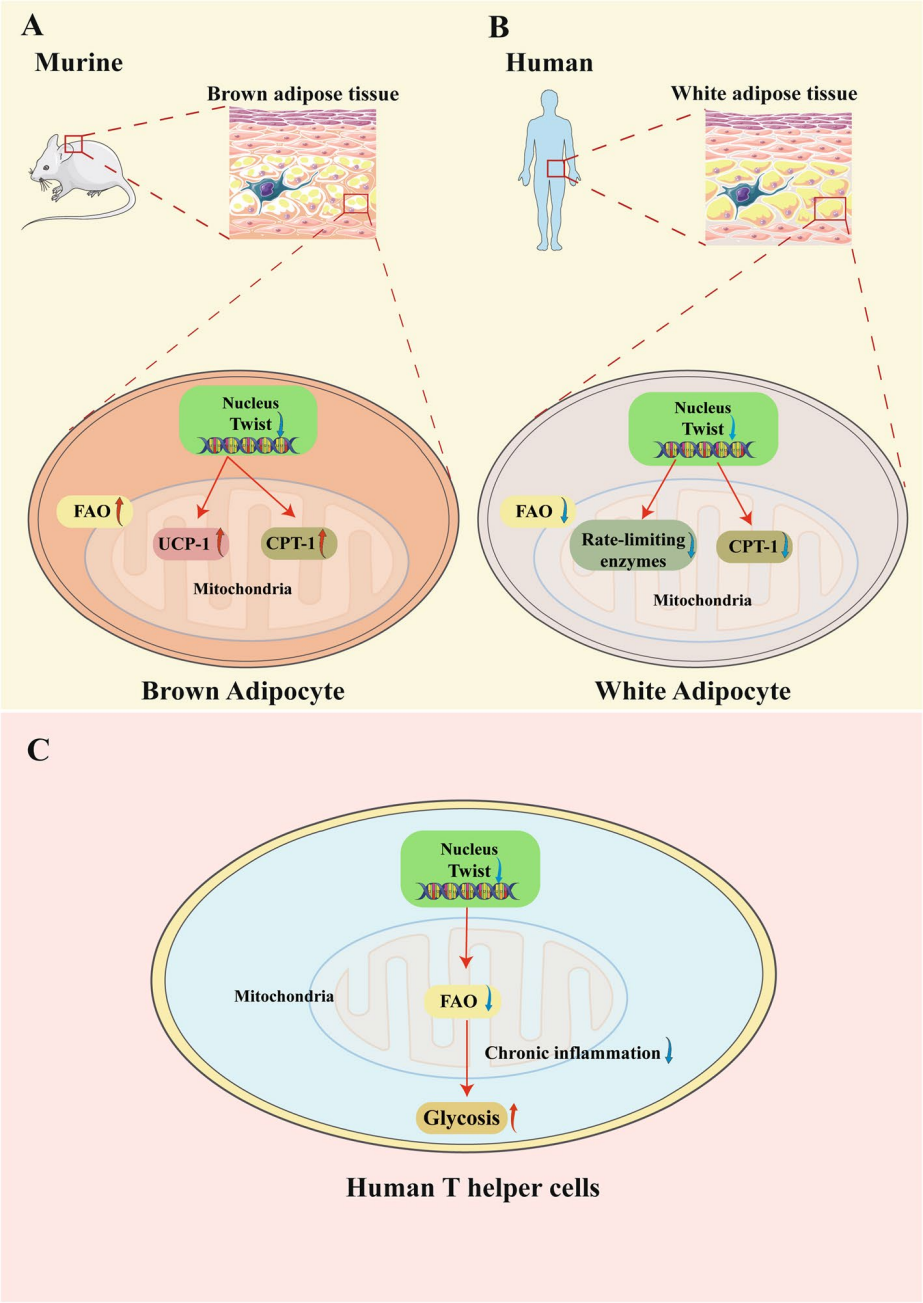
Roles of Twist1 in FAO in BAT, WAT and T helper cells. **A** In murine brown adipocytes, Twist1 knockout induced the expression of genes implicated in oxidative metabolism and FAO. **B** Twist1 knockdown in human differentiated white adipocytes reduced FAO, the rate-limiting enzymes for FAO and the mRNA level of CPT-1. **C** Twist1 is selectively expressed in the repeatedly activated murine Th1 cells and Twist1 acts as a crucial role in chronic inflammation and FAO in T helper cells

In murine brown adipocytes, Twist1 knockout induced the expression of genes implicated in oxidative metabolism and FAO, such as UCP-1 and CPT-1 [27, 49] (Fig. 1)*.* Twist1 transgenic mice on high fat diet (HFD) showed decreased expression of UCP-1 and FAO genes in BAT [25]*.* Similarly, Twist1 overexpression in C2C12 myotubes abolished the induction of PGC-1α-stimulated FAO without affecting basal oxygen consumption [28]*.* In humans Twist1 knockdown in differentiated white adipocytes reduced FAO, mRNA level of PGC-1α and CPT-1, and the rate-limiting enzymes for FAO without altering adiponectin, lipolysis, or glucose transportation [25]*.* As a pivotal regulator in brown adipocytes, PGC-1α overexpression in human white adipocytes can stimulate the browning process of WAT by increasing FAO and oxygen consumption [51]*.* The diverse results might attribute to the different sources of adipose tissue (BAT versus WAT). However, enormous evidence suggests that FAO only accounts for a little proportion of total metabolic pathways in white adipocytes [52], thus the effects of Twist1 on this process may be less important and remain to be determined.

Twist1 is also associated with the FAO of T helper cells (Fig. 1). Twist1 is highly abundant in CD4^+^ PD-1^+^ T cells in the inflamed synovia, which is established as a marker of chronic inflamed human tissues [53]*.* Twist1 is selectively expressed in the repeatedly activated murine Th1 cells instead of other types of T helper cells or the Th1 cells that are only activated once, indicating that Twist1 is required for the survival of repeatedly stimulated Th1 cells through stimulating FAO. This suggests a crucial role of Twist1 in chronic inflammation and FAO in T helper cells. Additionally, Th1 cells from Twist1-deficient mice manifested an increase in glycolysis compared to the cells from wild-type mice, suggesting an inhibitory role of Twist1 in glycolysis [29]*.* In humans, PD-1^+^ Th1 cells isolated from the synovial fluid of patients with juvenile idiopathic arthritis (JIA) also relied on FAO for survival. Silencing Twist1 in repeatedly activated Th1 lymphocytes freed the cells from Twist1-mediated FAO and made it possible to survive on glycolysis. Hence, ample evidence has shown that Twist1 is a key regulator of T helper cells metabolism in chronic inflammation via inhibiting glycolysis, constraining immunopathology, and stimulating FAO.

**Fig. 2:**
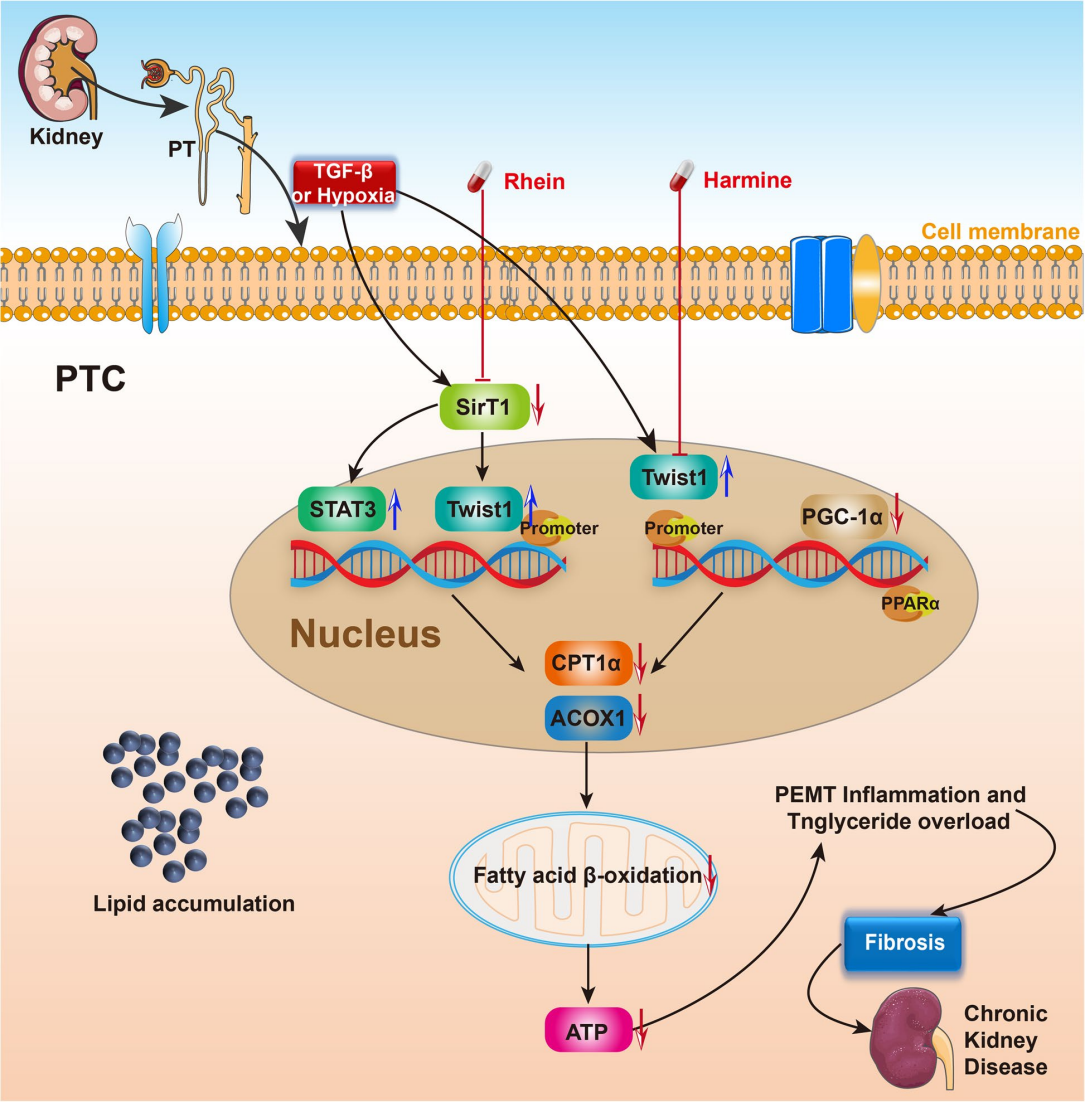
Twist1 promotes renal fibrosis by regulating fatty acid oxidation (FAO). Twist1 regulates FAO through interacting with CPT1α and PGC-1α in adipose tissue and renal tubular cells, and acts as a key regulator of chronic inflammation and FAO in T helper cells. Administration of Rhein or Harmine could downregulate Twist1 expression thus rescue the development of kidney fibrosis

In addition to lymphocytes, Twist1 has important impacts on the FAO in renal tubular cells (RTEs) and proximal tubular cells (PTCs). It has been a consensus that the disorders of FAO in PTCs play a necessary role in the development of renal fibrosis [39, 40]. The energy requirement in PTCs is in voracious need owing to the mass content of mitochondria, therefore, FAO becomes an ideal energy source for PTCs as FAO produces more ATP than glucose oxidation. Under the condition of FAO disorder in PTCs, descended FAO and ascended intracellular lipid droplets were identified together with the impaired mitochondrial function and increased levels of profibrogenic factors, which could cause the epithelial-to-mesenchymal transition (EMT), tubulointerstitial fibrosis (TIF), and progression to chronic kidney disease (CKD). In RTEs, Twist1 expression was significantly upregulated by TGF-β induction. Twist1 functions as upstream of CPT1α and causes FAO dysfunction by inhibiting the transcription of CPT1α, thus leading to EMT in RTEs. These negative effects could be reversed by rhein, an active anthraquinones isolated from a widely used traditional Chinese medicine rhubarb that has purgative effects, by inhibiting the expression and activity of Twist1. Mechanistically, rhein prevents the upstream SirT1/STAT3 signaling of Twist1 from activating the transcription of Twist1 in TGF-β-induced cells (Fig. 2). The consequent studies have demonstrated that Twist1 transcription is modulated by SirT1/STAT3 pathway, and that Twist1 is essential for CPT1α-mediated FAO dysfunction in RTEs [54]*.* Recently our group found that Twist1/PGC-1α axis also took a part in the downregulation of functional genes involved in fatty acid metabolism [30]. Hypoxia-induced upregulation and overexpression of Twist1 significantly decreased the levels of PGC-1α and its downstream target genes PPARα, CPT1, and peroxisomal acyl-coenzyme A oxidase 1 (ACOX1), leading to ATP depletion, triglyceride overload, and lipotoxicity-induced TIF. In a mouse model of kidney fibrosis, Twist1-specific knockout in PTCs stimulated PGC-1α expression and prevented the activation of mitochondrial FAO dysfunction. Administration of Twist1 inhibitor Harmine rescued the expression of PGC-1α and the genes that regulated fatty acid metabolism (PPARα, CPT1, and ACOX1), and inhibited the lipid aggregation in PTCs [30] (Fig. 2)*.* In brief, Twist1 regulates FAO through interacting with CPT1α and PGC-1α in adipose tissue and renal tubular cells and acts as a key regulator of chronic inflammation and FAO in T helper cells (Fig. 1). However, it is uncertain whether other transcription factors or signal pathways could interact with Twist1 to regulate FAO. There is ascending demand to examine a variety of potential Twist1 antagonists such as rhein and determine their functions on Twist1 mediated fatty acid oxidation as well as TGF-β/SirT1 pathway.

### Twist1 in glucose metabolism

The glucose uptake is dramatically increased in cancer cells, and Twist1 is inextricably intertwined with glucose metabolism. The functions of Twist1 have been studied in tumor tumorigenesis, stemness, progression, metastasis, and vasculogenic mimicry (VM), which is closely related to the Warburg effect of tumor metabolism. In hepatocellular carcinoma (HCC), for instance, Twist1 overexpression activated the pentose phosphate pathway, glycolysis, and several other metabolic pathways [32]. Twist1 transcriptionally activated Thymidine Phosphorylase (TP), which promoted HCC metastasis and VM formation via the Warburg effect, alleviating the deteriorated tumor microenvironment and bolstering tumor progression and metastasis [32]*.* In pancreatic ductal adenocarcinoma (PDAC), Twist1 acted as a crucial regulator of aerobic glycolysis and the Warburg effect by increasing the transcriptional expression of several glycolytic genes SLC2A1, HK2, ENO1, and PKM2 independent of HIF-1α or c-Myc [55]*.* Moreover, Twist1 overexpression increased the glycolysis pathway and the expression levels of the genes involved in glucose metabolism (e.g., PKM2, LDHA, and G6PD) in MCF10A mammary epithelial cells, and the gain-function of Twist1 also decreased mitochondrial mass, which could be magnified under the condition of hypoxia. Mechanistically, Twist1 promotes Warburg effect by contributing to the reprogramming of glucose metabolism and cell migration in breast cancer cells via the activation of the β1-integrin/FAK/PI3K/AKT/mTOR pathway and the inhibition of the p53 pathway by directly binding to the E-box of p53 [56]*.* In another study about Non-Small Cell Lung Cancer (NSCLC), Twist1 was established as a core component of PI3K/AKT/Twist1 pathway through which the recombinant total flavonoid aglycones extract significantly inhibited the glycolytic pathway and EMT of A549 cells [57]*.* However, more experimental evidence is needed to determine whether the tumor cell proliferation could be regulated by the gain/loss of function of Twist1.

Skeletal muscle is required for glucose homeostasis and is responsible for major glucose uptake from the blood. Although Twist1 has been found in skeletal muscle, the expression of Twist1 in the skeletal muscle is unchanged under the situation of obesity, T2D, and exercise training. Intriguingly, Twist1 overexpression led to the activation of pyruvate dehydrogenase and the increased glucose flux into the citric acid cycle rather than glycogen synthesis without impacting glucose uptake, which suggested that Twist1 could stimulate the glycogen utilization rate, one of the kernel characteristics of cancer development. Furthermore, overexpression of Twist1 in skeletal muscle increased the expression of inflammatory genes, promoted glucose utilization and cell growth pathways, and prohibited fatty acid synthesis without affecting FAO. Since the glucose utilization is increased whereas FAO is unchanged, it is likely that the total glucose metabolism is increased correspondingly. Nevertheless, the roles of Twist1 in skeletal muscle remain obscure as the expression level of Twist1 in skeletal muscle was unaltered in the patients with metabolic disorders. In short, Twist1 promotes tumor progression associated with the Warburg effect through activating pentose phosphate pathway and glycolysis as well as inhibiting the p53 pathway. Twist1, in skeletal muscle, not only decreases the fatty acid synthesis, but also increases inflammation and glycogen utilization. Considering that Twist1 upregulates the inflammation and downregulates fatty acid synthesis in skeletal muscle in a FAO-independent manner, it is rational to hypothesize that the functions of Twist1 depended on the cell differentiation stage, tissue specificity, and status quo of certain physiological environment [58]*.*

## Conclusion and perspectives

A profusion of studies concerning the functions of Twist1 in lipid / glucose metabolism are attention-getting and inspiring a mounting number of researchers to investigate more. Twist1, a bHLH transcriptional factor that is highly conserved in humans, is well known as its function in embryo development, organogenesis, tumorigenesis, tumor progression, metastasis, stemness, and vasculogenic mimicry. Twist1 is mainly expressed in adipose tissue and is implied to be associated with obesity, insulin resistance, inflammation, and the mitochondrial oxidative metabolism of adipose tissue by regulating transcriptional factors and cytokines such as PPARγ, PPARα, ERRα, NRF1, PGC-1α, TNF-α, IL-1β, and IL-6. Twist1 could be one of the key factors controlling inflammatory signals in human adipocytes. To date, the controversial results suggest a novel idea that the role of Twist1 in adipose tissue may differ depending on the species and the status quo. It is still unclear whether Twist1 directly interacts with the known signal pathways associated with inflammation, such as NF-κB pathway and JNK pathway. Therefore, further research is still needed to determine the precise mechanism by which Twist1 regulates the inflammatory factors in WAT. It is unknown how Twist1 regulates brown adipose metabolism, thus the Metabolic Chamber assay could be used to measure the in vivo energy expenditure. The roles of more microRNAs are to be identified in regulating Twist1 mediated brown adipose metabolism. Moreover, the evidence is desired to understand the mechanisms by which Twist1 regulates PPARγ expression as well as WAT browning. By suppressing FAO through multiple pathways, Twist1 mediates the genesis of tubular interstitial fibrosis to facilitate the progression of chronic kidney disease. It is urgent to examine a variety of potential Twist1 antagonists such as rhein or Harmine, and determine their functions on Twist1 mediated FAO as well as TGF-β/SirT1 pathway and the other pathways using two-hybrid and microarray assays. In glucose metabolism, Twist1 participates in the cancer progression by promoting Warburg effect and modulating glucose metabolism such as pentose phosphate pathway and glycolysis. However, it is not clear whether the proliferation of tumor cells could be regulated by the gain/loss of function of Twist1, which could be tested by the treatment of the agonists or antagonists of Twist1. These studies provide the possible strategy to treat metabolic disorders, tumorigenesis, and fibrotic diseases, but more studies are still intensely needed to further elucidate the underlying mechanisms. In conclusion, this review discusses the functional roles and underlying mechanisms of Twist1 in lipid and glucose metabolism and may shed a light on considering Twist1 as a potential target for clinical therapy.

## Data Availability

Not applicable.

## Abbreviations

bHLH: Basic helix-loop-helix
IR: Insulin resistance
PGC-1α: Peroxisome proliferator-activated receptor-γ coactivator
PPAR-δ: Peroxisome proliferator activated receptorfatty acid oxidation (FAO)
TECs: Tubular epithelial cells
TIF: Tubulointerstitial fibrosis
WAT: White adipose tissue
BAT: Brown adipose tissue
JNK: C-Jun N-terminal kinase
TNF-α: Tumor necrosis factor-α
IL-6: Interleukin-6
MCP-1: Monocyte chemotactic protein-1
HOMA-IR: Homeostasis model assessment of insulin resistance
SAT: Subcutaneous adipose tissue
VAT: Visceral adipose tissue
HFD: High fat diet
BAs: Brown adipocytes
UCP1: Mitochondrial uncoupling protein 1
JIA: Juvenile idiopathic arthritis
RTEs: Renal tubular cells
PTCs: Proximal tubular cells
CKD: Chronic kidney disease
ACOX1: Peroxisomal acyl-coenzyme A oxidase 1
VM: Vasculogenic mimicry
HCC: Hepatocellular carcinoma
TP: Thymidine Phosphorylase
PDAC: Pancreatic ductal adenocarcinoma
NSCLC: Non-Small Cell Lung Cancer
